# Evaluation of fruit phenotypic diversity in 210 *Malus sieversii* germplasm resources

**DOI:** 10.3389/fpls.2026.1793526

**Published:** 2026-04-13

**Authors:** Bian Ran, Ling Zhu, Shengjun Zhang, Junnan Jian, Fang Tian, Shilei Dong, Shimin Tang, Xuechao Zhang

**Affiliations:** Institute of Agricultural Sciences of Ili Kazakh Autonomous Prefecture, Key Laboratory of Crop Breeding and Quality Testing, Yili Prefecture Agriculture and Rural Affairs Bureau, Yili, Xinjiang, China

**Keywords:** diversity, fruit, germplasm resources, *Malus sieversii*, phenotype

## Abstract

This study utilized 210 *Malus sieversii* germplasm fruit samples collected from seven natural populations, which exhibit rich genetic diversity in their hereditary traits. The research findings systematically evaluated and identified the genetic diversity of 31 fruit phenotypic traits using five statistical analysis methods: frequency analysis, quantitative trait statistical analysis, correlation analysis, principal component analysis (PCA), and cluster analysis, thereby enabling the screening of distinctive germplasm resources. Frequency distribution analysis revealed that a high proportion of the samples had fruit weight ranging from 10–20 g, fruit longitudinal diameters from 25–31 mm, fruit transverse diameters from 30–39 mm, pedicel length from 10–20 mm, and soluble solids content (SSC) from 11.4%-13.75%. The fruit shape of *M. sieversii* is predominantly “oblate” or “oblique”, with the ground color mostly “green-yellow”, flesh color mostly “green-white”, and flavor predominantly “sour”. Statistical analysis of quantitative traits revealed that the coefficient of variation for eight quantitative traits ranged from 7.02%-38.24%. Among these, fruit weight and pedicel length exhibited relatively high coefficients of variation, indicating rich genetic diversity. Correlation analysis showed that fruit weight was significantly positively correlated with fruit longitudinal diameters, fruit transverse diameters and pedicel diameter, while pedicel length was significantly negatively correlated with pedicel diameter. Fruit ground color showed significant negative correlations with flesh color and juiciness. PCA extracted 12 principal components with a cumulative contribution rate of 68.43%. Among these, the first four principal components - fruit size, flavor quality, basin morphology, and pedicel characteristics - were identified as the main dimensions constituting the phenotypic diversity of *M. sieversii* fruits. The 210 germplasm resources were classified into four clusters: Cluster I comprised small-fruit, high-acidity resources; Cluster II, resources with long pedicel traits; Cluster III, oblate-shaped, short-pedicel resources; and Cluster IV, specific resources characterized by large fruit size and favorable flavor quality. The results provide a theoretical basis for the effective utilization of *M. sieversii* germplasm resources and offer references for the selection and breeding of specific resources.

## 1 Introduction

*Malus sieversii* (Ledeb.) M.Roem., a species within the genus Malus of the Rosaceae family and also known as Sievers’ apple or Tianshan apple, is a Tertiary relict species (Li, 2001) characterized by high homology and primitiveness (Duan et al., 2017), making it a highly valuable germplasm resource. Based on fruit traits, *M. sieversii* in the Ili region has been classified into 84 distinct types (Lin and Cui, 2000), with the coefficients of variation for characteristics such as fruit shape, color, size, and stalk length exceeding 10% (Feng et al., 2006). Significant genetic diversity has been observed in the fruit morphology, flavor (Forsline et al., 2003), and the fruit quality of four superior lines of *M. sieversii* (Yu et al., 2017), indicating promising selection potential.

The fruit biological traits of *M. sieversii* exhibit considerable diversity. Quantitative traits, including fruit weight, fruit longitudinal and transverse diameters, SSC, pedicel length and diameters, demonstrate substantial genetic variation in populations from Xinyuan and Gongliu counties of the Ili region, with coefficients of variation (CV) exceeding 10% (Wang et al., 2016). Among 41 accessions of *M. sieversii* germplasm resources, significant variation was observed in quantitative fruit traits, with fruit weight ranging from 4.3-67.5 g and a CV as high as 63% (Diao et al., 2019). Analysis of 117 accessions revealed broad genetic variability across 16 fruit quality indicators. Notably, the CV for the red-green chromatic difference of peel color reached 268.29%, while peeled firmness ranged from 4.47-19.47 kg/cm² with a CV of 31.97% (Liu, 2023). Significant differences were observed in fruit-related traits among 42 *M. sieversii* accessions, with fruit weight showing a high CV of 43.23%, and pedicel length varying between 11.9-37.78 mm (CV: 25.61%) (Su, 2025). While numerous studies have been conducted on the biological characteristics of Malus sieversii fruits, these investigations have generally been limited by small sample sizes and a narrow selection of fruit traits, lacking representation across large populations. This study systematically and comprehensively evaluated the fruit traits of 210 Malus sieversii germplasm resources from seven natural populations using frequency statistics, principal component analysis, and cluster analysis across 31 fruit trait indicators. Using frequency statistics, PCA, and cluster analysis, the research aims to provide a theoretical foundation for the effective utilization of these resources and to identify potential parent materials for the breeding of distinctive varieties.

## 2 Materials and methods

### 2.1 Experimental materials

The test materials consisted of fruits from 210 accessions of *M. sieversii* germplasm resources. All fruits were harvested from the National Wild Apple Germplasm Resource Orchard (Ili)(Central coordinates: North 43°55.8435′, East 81°23.2040′), where uniform field management practices were applied. Fruits were collected at the early ripening stage, ensuring consistent maturity, uniform size, and the absence of pests and diseases. After harvest, the fruits were transported to the laboratory, and all trait measurements were conducted on the same day. For each accession, five fruits were randomly selected per replicate, with three replicates performed. The tested *M. sieversii* germplasm resources originated from seven natural populations located in Huocheng, Gongliu, Xinyuan, Chabuchaer, Yining County, Tuoli, and Emin. Specifically, the populations included 26 accessions from Huocheng, 9 from Chabuchaer, 33 from Yining County, 63 from Gongliu, 34 from Xinyuan, 14 from Tuoli, and 31 from Emin. The detailed accession numbers are listed in **Table 1**.

**Table 1:** Numbers and sources of 210 *M. sieversii* germplasm resources.

Population	Number	Germplasm accession name
Huocheng	26	HG1、HG2、HG3、HDM5、HDM11、HDM12、HDM13、HDM14、HDM15、HDM16、HDM17、HDM19、HDM20、HDM21、HDM22、HDM27、HDM29、HDM30、HDM34、HDM35、HDM37、HDM39、HDM41、HDM42、HDM44、HDM47
Chabuchaer	9	CK3、CK4、CK7、CK8、CK9、CK10、CK16、CK18、HONG3
Yining County	33	YA1、YA3、YA4、YA6、YA7、YA8、YA9、YA12、YA13、YA14、YA15、YA16、YA17、YA18、YA19、YA32、YA33、YA34、YA36、YA41、YA42、YG1、YG2、YG4、YG5、YG13、YG14、YG17、YG19、YJ21、YJ22、YJ23、YJ24
Gongliu	63	GALS1、GALS2、GALS3、GALS4、GB2、GB5、GB6、GB7、GB8、GB9、GB10、GB12、GB13、GB15、GB17、GB18、GB19、GB20、GB21、GB22、GB23、GB25、GD5、GD9、GD10、GD11、GD12、GD13、GD15、GD17、GD18、GD25、GH1、GH3、GH5、GJS2、GJS3、GJS4、GJS5、GJS6、GJS8、GJS9、GJS10、GJS11、GJS14、GJS15、GJS16、GJS17、GJS18、GJS19、GJS20、GJS23、GJS24、GJS25、GJS26、GJS27、GJS29、GJS30、GK3、GQ1、GX1、GX2、GX4
Xinyuan	34	Ye Pingguo Wang、XY1、XY2、XY4、XY6、XY7、XY8、XY9、XY10、XY12、XY17、XY20、XY21、XY23、XY26、XY27、XY28、XY29、XY30、XY34、XY42、XY43、XY51、XY54、XY57、XY58、XY61、XY62、XY69、XY79、XY82、XY84、XY87、XY92
Tuoli	14	LF3、LF4、LF6、LF9、LF10、LF13、LF14、LF16、LF19、LF20、LF22、LF24、LF25、LF29、
Emin	31	EM3、EM4、EM5、EM6、EM8、EM9、EM21、EM11、EM13、EM23、EM25、EM26、EM27、EM30、EM31、EM42、EM46、EM51、EM52、EM53、MY1、ML2、ML11、ML12、ML13、ML14、ML15、ML16、ML22、ML24、ML29

### 2.2 Experimental methods

Phenotypic characterization of the fruit from *M. sieversii* germplasm resources was conducted following the (*Descriptors and Data Standard for Apple* (*Malus* spp. *Mill.*) ) (Wang et al., 2005). The investigation included a total of 31 traits, comprising eight quantitative traits: pedicel length and diameter, fruit longitudinal and transverse diameters (measured with a vernier caliper, accuracy 0.01 mm), and fruit index (longitudinal diameters/transverse diameter ratio), fruit weight (measured with an electronic balance, accuracy 0.01 g), flesh firmness (measured using a GY-4 firmness tester, accuracy 0.01 kg/cm²), SSC (determined with a PAL-BX/ACID 5 sugar-acid analyzer). Twenty-three descriptive traits (**Table 2**): stalk cavity depth, stalk cavity width, stem-end russet coverage, sepal condition, sepal posture, calyx basin depth, calyx basin width, calyx-end russet coverage, fruit russeting severity, bloom, cuticular smoothness, fruit rib, fruit shape, ground color, lenticel diameter, lenticel density, core diameter, locules state, flesh color, flesh texture, fruit juice, fruit flavor, comprehensive internal quality evaluation.

**Table 2:** Assignment of fruit descriptive traits for *M. sieversii* germplasm resources.

Traits	Assignment	Traits	Assignment
Stalk cavity depth	1 = Shallow, 2 = Medium, 3 = Deep	Fruit shape	1 = Subglobose, 2 = Oblate, 3 = Ellipsoid, 4 = Ovate, 5 = Cylindrical, 6 = Oblique
Stalk cavity width	1 = Narrow, 2 = Medium, 3 = Broad	Ground color	1 = Pale Green, 2 = Yellow-Green, 3 = Green, 4 = Green-Yellow, 5 = Yellowish White, 6 = Pale Yellow, 7 = Yellow
Stem-end russet coverage	1 = None, 2 = Slight, 3 = Moderate, 4 = Extensive	Lenticel diameter	1 = Small, 2 = Medium, 3 = Large
Sepal condition	1 = Persistent, 2 = Remnant, 3 = Deciduous	Lenticel density	1 = Sparse, 2 = Medium, 3 = Dense
Sepal posture	1 = Reflexed, 2 = Erect, 3 = Convergent	Core diameter	1 = Small, 2 = Medium, 3 = Large
Calyx basin depth	1 = Shallow, 2 = Medium, 3 = Deep	Locules state	1 = Closed, 2 = Semi-Open, 3 = Fully Open
Calyx basin width	1 = Narrow, 2 = Medium, 3 = Broad	Flesh color	1 = White, 2 = Yellowish White, 3 = Pale Yellow, 4 = Yellow, 5 = Greenish White, 6 = Yellow-Green, 7 = Light Red
Calyx-end russet coverage	1 = None, 2 = Slight, 3 = Moderate, 4 = Extensive	Flesh texture	1 = Soft and Spongy, 2 = Mealy, 3 = Crisp and Tender, 4 = Firm and Crisp, 5 = Hard
Fruit russeting severity	1 = None, 2 = Slight, 3 = Moderate, 4 = Extensive	Fruit juice	1 = Low, 2 = Medium, 3 = High
Bloom	1 = Absent, 2 = Present	Fruit flavor	1 = Mildly Sweet, 2 = Sweet-Sour, 3 = Balanced Sweet-Sour, 4 = Sour-Sweet, 5 = Sour, 6 = Very Sour
Cuticular smoothness	1 = Rough, 2 = Moderately Smooth, 3 = Smooth	Comprehensive internal quality evaluation	1 = Poor, 2 = Fair, 3 = Good, 4 = Excellent
Fruit rib	1 = Absent, 2 = Present		

### 2.3 Data analysis

Data on fruit phenotypic characteristics were organized and classified using Microsoft Excel 2019, and frequency distribution plots were generated. Statistical analysis of quantitative fruit traits and PCA were performed using SPSS 22. Correlation analyses were conducted separately for quantitative traits and for phenotypic traits. A correlation heatmap was created using the Hiplot online platform. K-means cluster analysis was performed using Origin software.

## 3 Results analysis

### 3.1 Frequency distribution of fruit phenotypic characteristics

#### 3.1.1 Pedicel and stalk cavity traits

**Figure 1:**
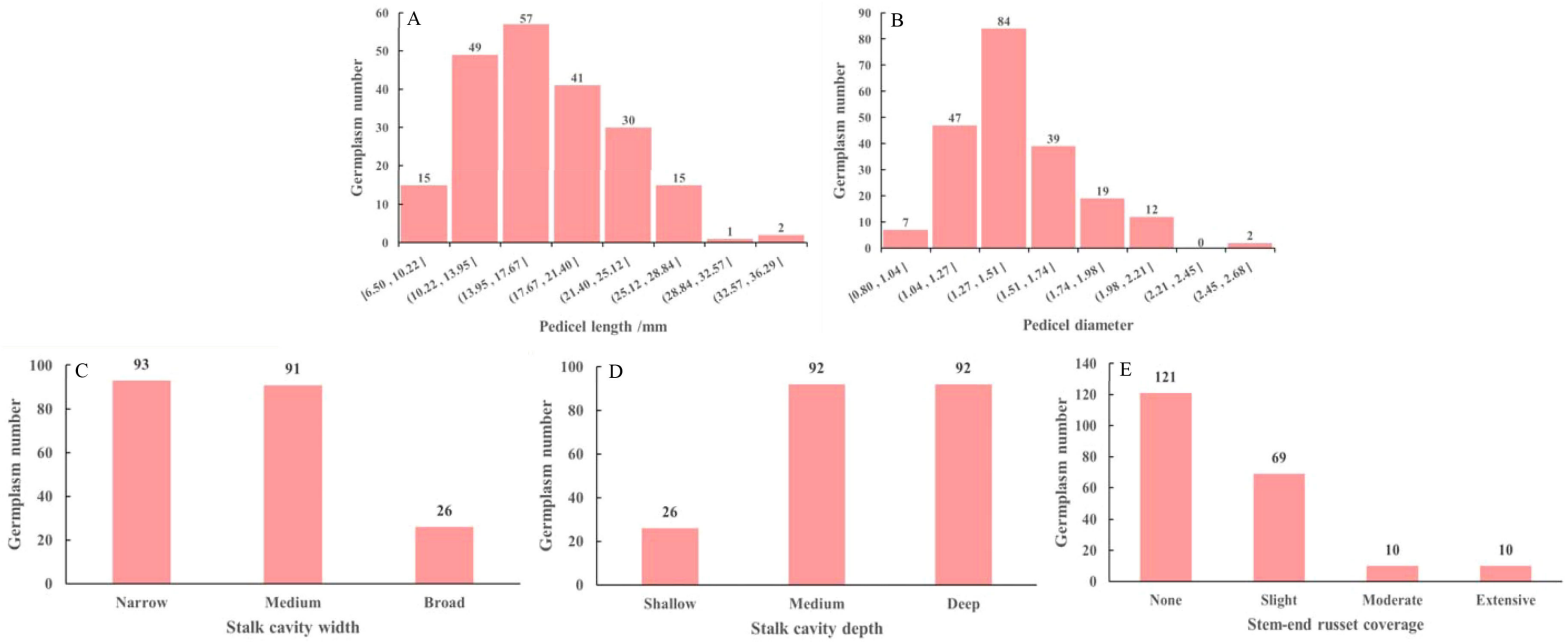
Pedicel length **(A)**, pedicel diameter **(B)**, stalk cavity width **(C)**, stalk cavity depth **(D)**, and stem-end russet coverage **(E)** of 210 *M. sieversii* germplasm accessions.

The pedicel and stalk cavity traits of the *M. sieversii* germplasm resources are shown in **Figure 1**. Among the 210 accessions, pedicel length ranged from 6.5 to 36.29 mm. Specifically, 14 accessions had pedicels shorter than 10 mm, 3 accessions had pedicels longer than 30 mm, and 135 accessions (64.28%) had pedicel lengths between 10 and 20 mm (**Figure 1A**). Pedicel diameter ranged from 0.8 to 2.68 mm across the accessions. 3 accessions had pedicel diameters less than 1 mm, 14 accessions had diameters greater than 2 mm, and 112 accessions (53.3%) fell within the 1.2-1.5 mm range (**Figure 1B**). Regarding stalk cavity width, the 210 accessions (44.28%) were classified as “narrow”, followed by “medium” (43.33%), with “broad” being the least common (12.38%) (**Figure 1C**). For stalk cavity depth, “deep” and “medium” were equally prevalent, each accounting for 43.8% of the total samples, while “shallow” was less common (12.38%) (**Figure 1D**). In terms of stem-end russet coverage, “none” was the most frequent category (57.62%), whereas “moderate” and “extensive” russeting were relatively rare (each 4.76%) (**Figure 1E**).

#### 3.1.2 Calyx traits of the fruit

**Figure 2:**
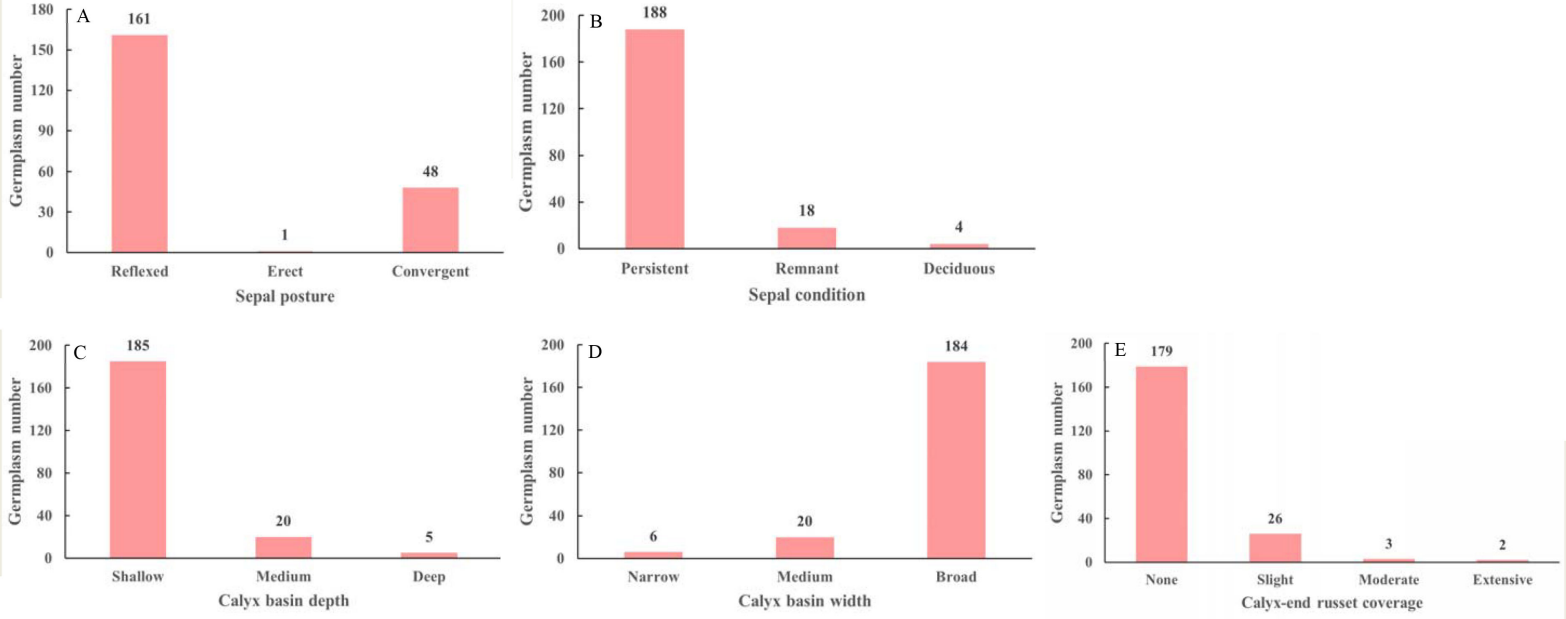
Sepal posture **(A)**, sepal condition **(B)**, calyx basin depth **(C)**, calyx basin width **(D)**, calyx-end russet coverage **(E)** of 210 *M. sieversii* germplasm accessions.

The calyx traits of the *M. sieversii* germplasm resources are shown in **Figure 2**. Among the 210 accessions, 161 (76.19%) exhibited a “reflexed” sepal posture, followed by “convergent” (22.85%), whereas “erect” was rare, observed in only one accession (0.48%) (**Figure 2A**). Regarding sepal condition, 188 accessions (89.52%) had “persistent” sepals, whereas a small proportion (1.9%) had “deciduous” sepals (**Figure 2B**). For calyx basin depth, the majority of accessions (88.09%) were classified as “shallow,” with only 5 accessions (2.38%) categorized as “deep” (**Figure 2C**). Similarly, for calyx basin width, “broad” was the most common trait (87.62%), while only 6 accessions (2.86%) were classified as “narrow” (**Figure 2D**). In terms of calyx-end russet coverage, most accessions (85.24%) had “none”, whereas “moderate” and “extensive” russeting were rare, observed in only 3 and 2 accessions, respectively (**Figure 2E**).

#### 3.1.3 Overall external fruit traits

**Figure 3:**
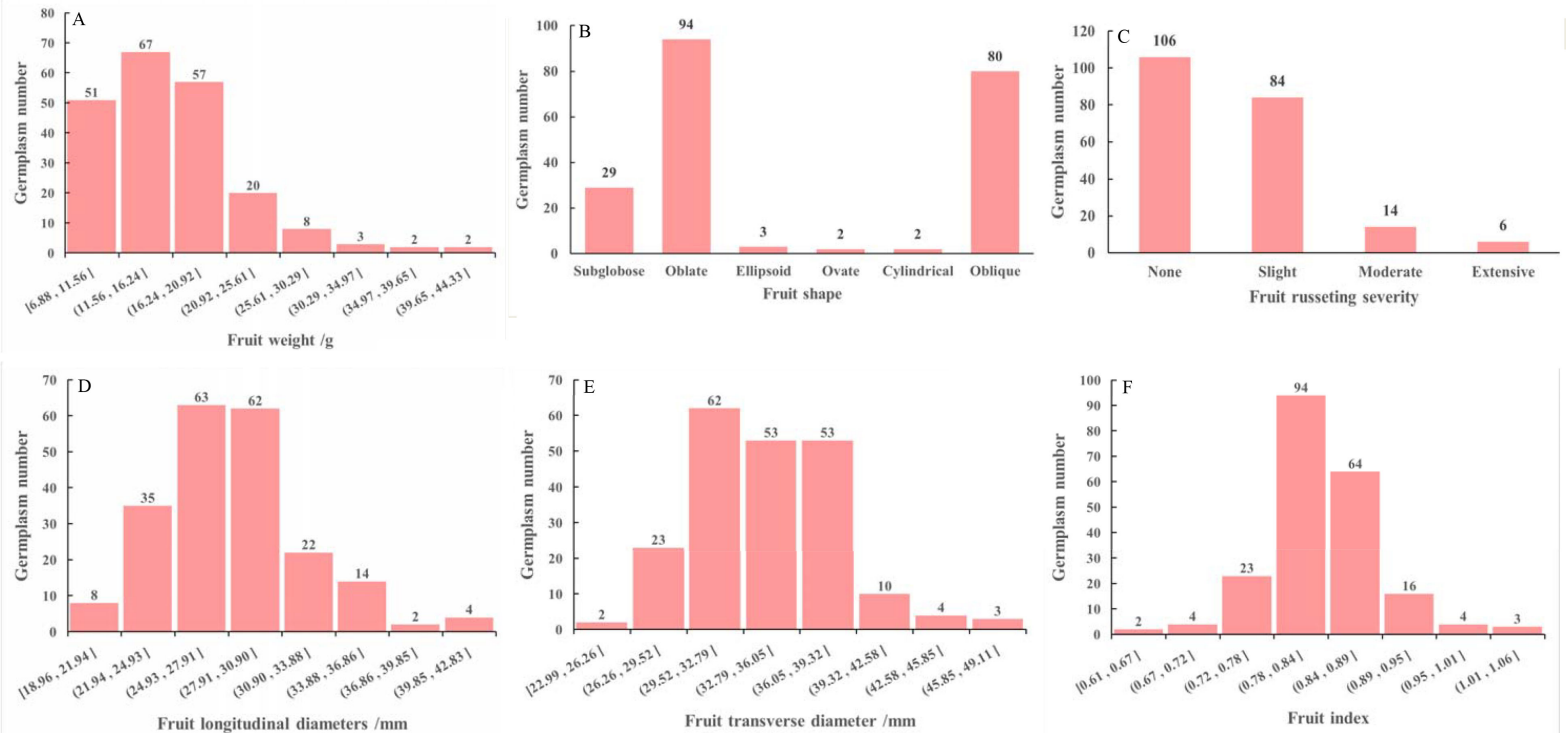
Fruit weight **(A)**, fruit shape **(B)**, fruit russeting severity **(C)**, fruit longitudinal diameters **(D)**, fruit transverse diameter **(E)** and fruit index (F) of 210 *M. sieversii* germplasm accessions.

The overall external traits of the *M. sieversii* germplasm resources are shown in **Figure 3**. Among the 210 accessions, 144 (68.57%) had fruit weight between 10 and 20 g, 16.67% were in the 20–30 g range, 24 accessions weighed less than 10 g, and 7 accessions exceeded 30 g (**Figure 3A**). In terms of fruit shape, “oblate” was the most prevalent form (44.76%), followed by “oblique” (38.09%). Shapes such as “ellipsoid”, “ovate”, and “cylindrical” were relatively rare, together accounting for only 3.33% of the total (**Figure 3B**). In terms of fruit russeting severity, “none” was the predominant category, observed in 106 accessions and accounting for 50.48% of the total. This was followed, in descending order, by “slight” (40%), “moderate” (6.67%), and “extensive” (2.86%) russeting (**Figure 3C**). Fruit longitudinal diameters ranged widely among accessions: 125 accessions (59.52%) measured between 25 and 31 mm, 20.48% were shorter than 25 mm, 14.29% fell within 31–36 mm, and only 8 accessions exceeded 36 mm (**Figure 3D**). For fruit transverse diameters, 156 accessions (74.29%) measured between 30 and 39 mm, 16.67% were narrower than 30 mm, and 9.04% were wider than 39 mm (**Figure 3E**). The fruit index for most accessions (164, or 78.10%) ranged from 0.78 to 0.90. Values between 0.60 and 0.72 were much less common, similar as between 0.95 and 1.06 (**Figure 3F**).

#### 3.1.4 Fruit peel traits

**Figure 4:**
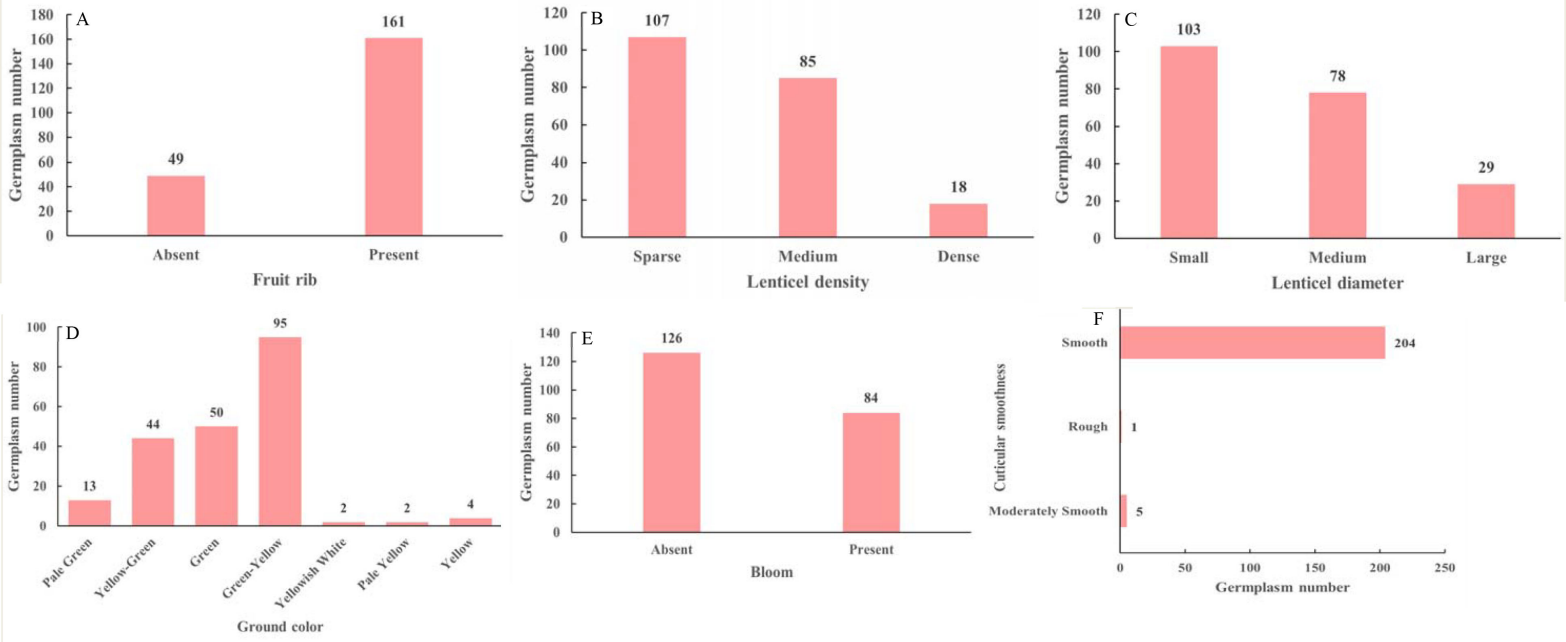
Fruit rib **(A)**, lenticel density **(B)**, lenticel diameter **(C)**, ground color **(D)**, bloom **(E)** and cuticular smoothness **(F)** of 210 *M. sieversii* germplasm accessions.

The fruit peel traits of the *M. sieversii* germplasm resources are shown in **Figure 4**. Among the 210 accessions, the concerning fruit ribbing, most accessions (76.67%) had ribbed fruit surfaces, whereas 23.33% “absent” (**Figure 4A**). For lenticel density, a higher proportion of accessions (50.95%) had “sparse”, while “dense” were less common (8.57%) (**Figure 4B**). Similarly, regarding lenticel diameter, “small” lenticels were more prevalent (49.05%), while “large” lenticels were relatively rare (13.81%) (**Figure 4C**). Predominant ground color was “green-yellow” (45.24%), followed by “green” (23.81%) and “yellow-green” (20.95%). Colors such as “yellowish white”, “pale yellow”, and “yellow” were less common (**Figure 4D**). Regarding bloom, a majority of the accessions (60%) showed “absent”, while the “presence” of bloom was less frequent (40%) (**Figure 4E**). In terms of cuticular smoothness, 97.14% of the accessions had “smooth”, 2.38% were “moderately smooth”, and only one accession (0.48%) exhibited “rough” (**Figure 4F**).

#### 3.1.5 Core and flesh traits

**Figure 5:**
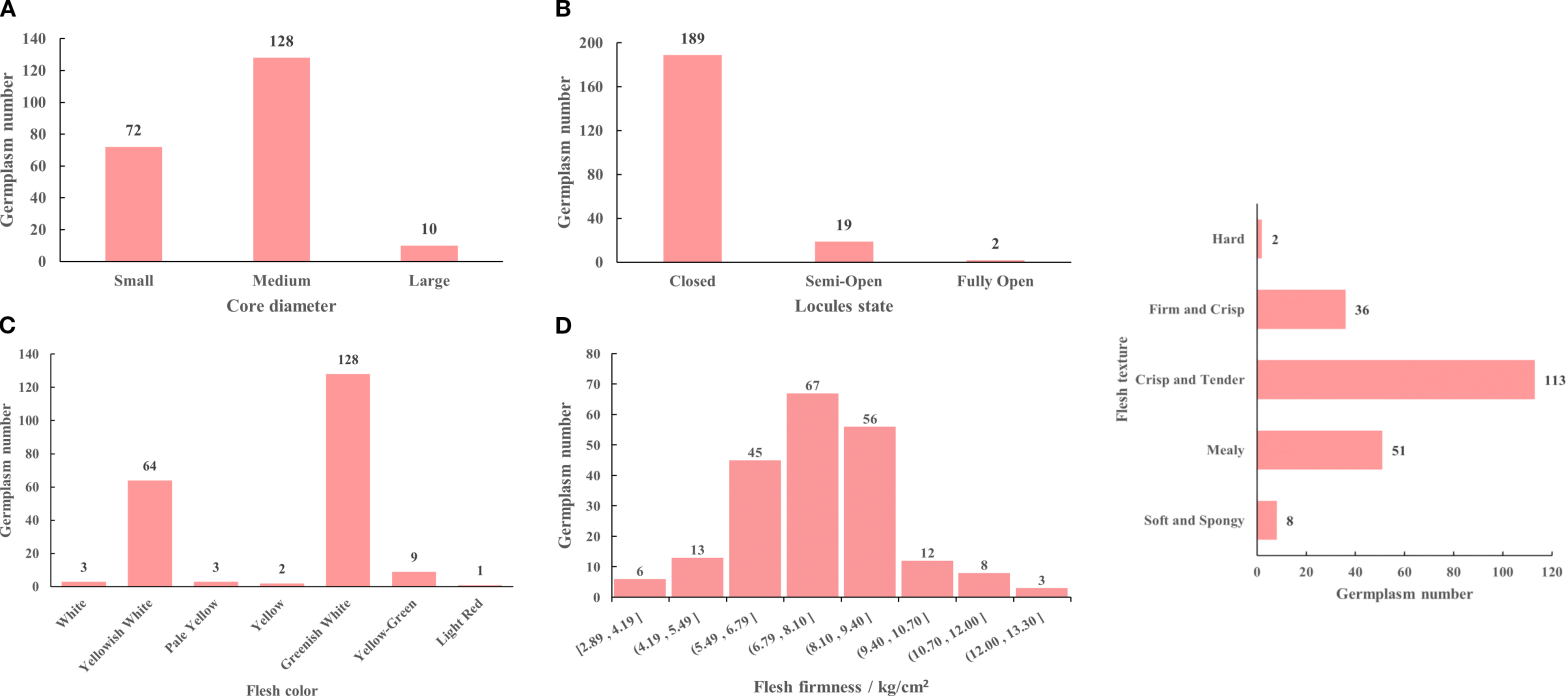
Core diameter **(A)**, locules state **(B)**, flesh color **(C)**, flesh firmness **(D)**, flesh texture **(E)** of 210 *M. sieversii* germplasm accessions.

The core traits of the *M. sieversii* germplasm resources are shown in **Figure 5**. Among the 210 accessions, core diameter was predominantly “medium” (60.95%), followed by “small”, while “large” cores were less common (4.76%) (**Figure 5A**). Regarding locule state, the majority of accessions (90%) had “closed” locules, 9.05% had “semi-open” locules, and only 2 accessions (0.95%) exhibited “fully open” locules (**Figure 5B**). Concerning flesh color, “greenish white” was the most prevalent (60.95%), followed by “yellowish white” (30.48%). Other colors, including “yellow-green”, “white”, “pale yellow”, “yellow”, and “light red”, were observed in descending order of frequency (**Figure 5C**). In terms of flesh firmness, 125 accessions (59.52%) had values between 6.8 and 9.5 kg/cm², 11 accessions (5.24%) were below 5 kg/cm², and 14 accessions (6.67%) exceeded 10 kg/cm² (**Figure 5D**). For flesh texture, “crisp and tender” was the dominant type (53.81%), followed by “mealy” (24.29%). Notably, only 2 accessions had “hard” flesh (**Figure 5E**).

#### 3.1.6 Fruit quality traits

**Figure 6:**
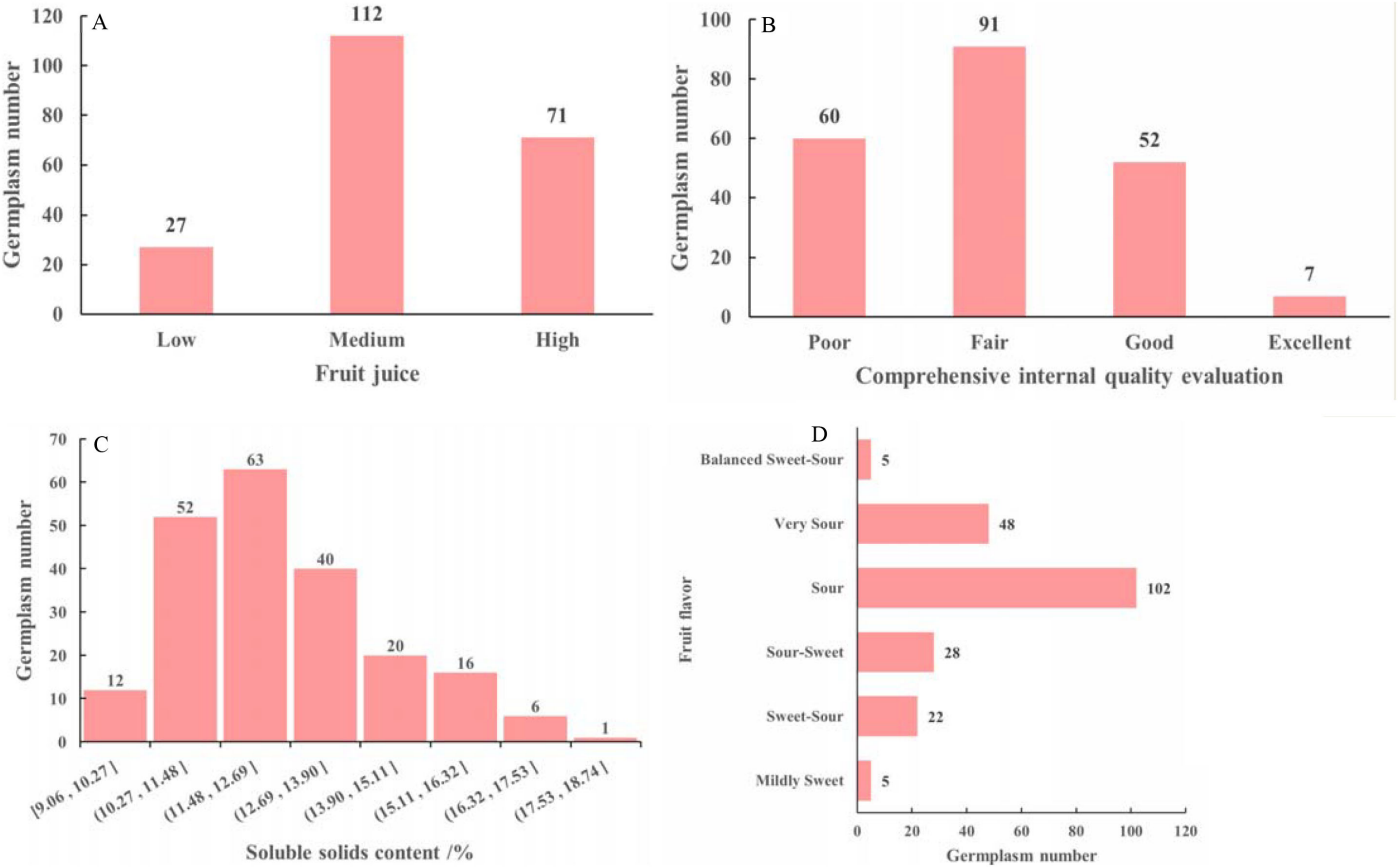
Fruit juice **(A)**, comprehensive internal quality evaluation **(B)**, soluble solids content **(C)**, fruit flavor **(D)** of 210 *M. sieversii* germplasm accessions.

The fruit quality traits of the *M. sieversii* germplasm resources are shown in **Figure 6**. Among the 210 accessions, fruit juice was predominantly “medium” (53.33%), followed by “high” juice (33.81%), while “low” juice was less common (12.86%) (**Figure 6A**). For the comprehensive internal quality evaluation, a “fair” rating was most frequent (43.33%), followed by a “good” rating (24.76%). Only 7 accessions (3.33%) received an “excellent” rating (**Figure 6B**). The SSC of the fruits ranged from 9.06%-20.77%. A higher proportion of accessions (47.62%) had SSC values between 11.4% and 13.75%, while 24 accessions (11.43%) exceeded 15% (**Figure 6C**). Regarding fruit flavor, “sour” was the most prevalent category (48.57%), followed by “very sour” (22.86%). “Sweet-sour” and “sour-sweet” flavors were less common, accounting for 13.33% and 10.48%, respectively. “Mildly sweet” and “balanced sweet-sour” flavors were the least frequent, each representing only 2.38% of the accessions (**Figure 6D**).

### 3.2 Statistical analysis of fruit quantitative traits

Statistical analysis of the eight quantitative traits revealed significant differences (**Table 3**). The CV ranged from 7.02% to 38.24%. Fruit weight exhibited the highest CV (38.24%), followed by pedicel length (31.03%), indicating substantial variation and rich genetic diversity for these traits within the population. In contrast, fruit longitudinal and transverse diameters, and fruit index showed lower CVs, suggesting limited variation and relatively low genetic diversity for these characteristics.

**Table 3:** Statistical analysis of quantitative fruit traits in *M. sieversii* germplasm resources.

Traits	Min	Max	Mean	Standard deviation (SD)	Coefficient of variation(%) (CV)
Pedicel length (mm)	6.50	36.29	17.23	5.35	31.03
Pedicel diameter (mm)	0.80	2.68	1.47	0.29	20.01
Fruit longitudinal diameters (mm)	18.96	42.83	28.24	4.05	14.33
Fruit transverse diameter (mm)	22.99	49.11	34.05	4.27	12.55
Fruit index	0.61	1.06	0.83	0.06	7.02
Fruit weight (g)	6.88	44.33	16.12	6.16	38.24
Flesh firmness (kg/cm^2^)	2.89	13.30	7.60	1.71	22.49
SSC (%)	9.06	18.74	12.57	1.73	13.72

### 3.3 Correlation analysis of fruit traits

#### 3.3.1 Correlation analysis of fruit quantitative traits

**Figure 7:**
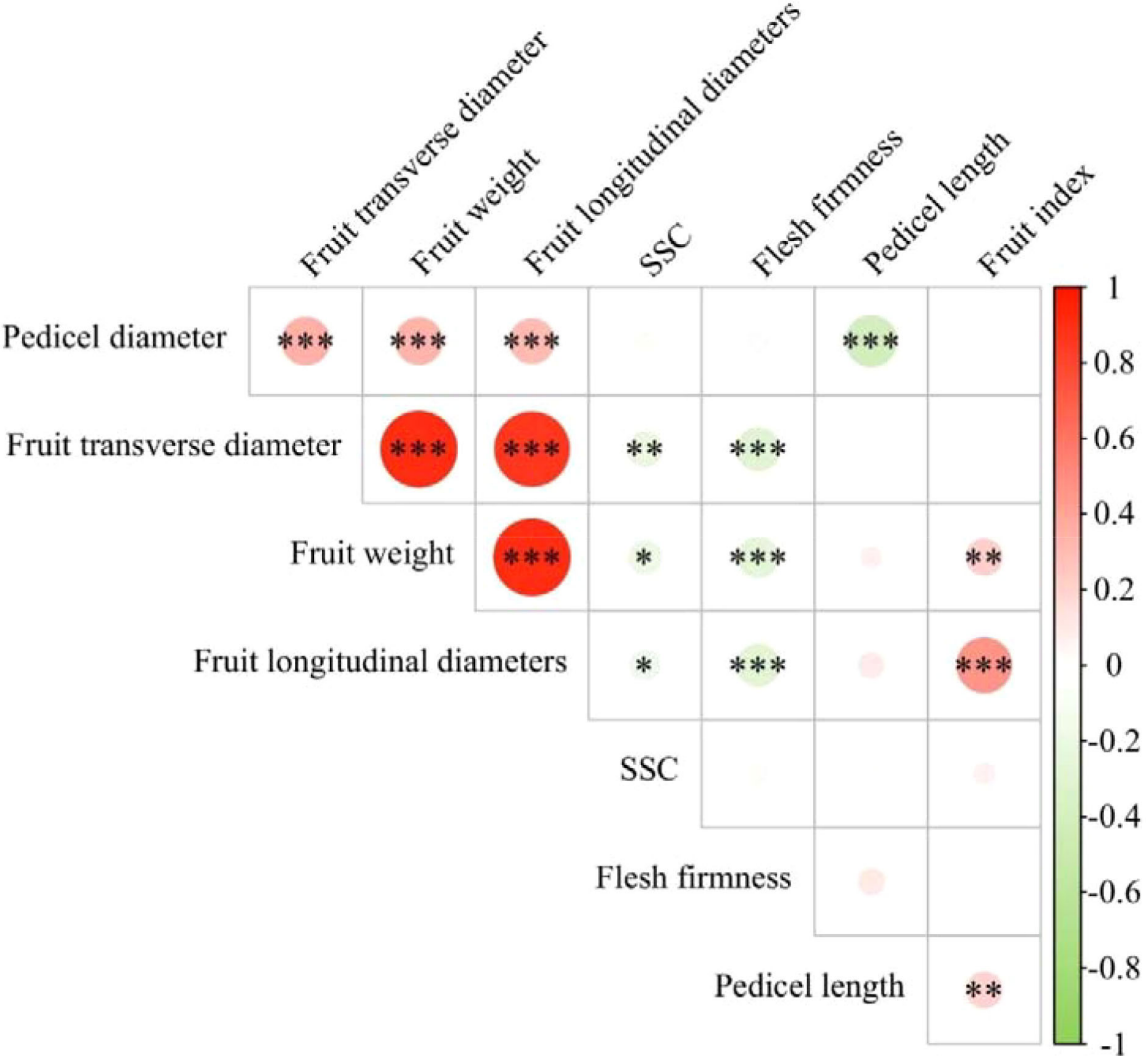
Correlation heatmap of fruit quantitative traits in *M. sieversii* germplasm resources. *:There are significant differences at the 0.05 level; **:There are significant differences at the 0.01 level; ****:There are significant differences at the 0.0001 level.

The correlation heatmap for the quantitative traits of *M. sieversii* germplasm resources is shown in **Figure 7**. Among the findings, fruit longitudinal diameters showed a highly significant positive correlation with fruit transverse diameters and fruit index, and a highly significant negative correlation with flesh firmness. Fruit weight exhibited highly significant positive correlations with fruit longitudinal and transverse diameters, and fruit index, while showing a highly significant negative correlation with flesh firmness. Fruit transverse diameters was significantly negatively correlated with both flesh firmness and SSC. Pedicel diameter demonstrated highly significant positive correlations with fruit weight, fruit longitudinal and transverse diameters. Pedicel length was significantly positively correlated with fruit index and significantly negatively correlated with pedicel diameter.

#### 3.3.2 Correlation analysis of fruit descriptive traits

**Figure 8:**
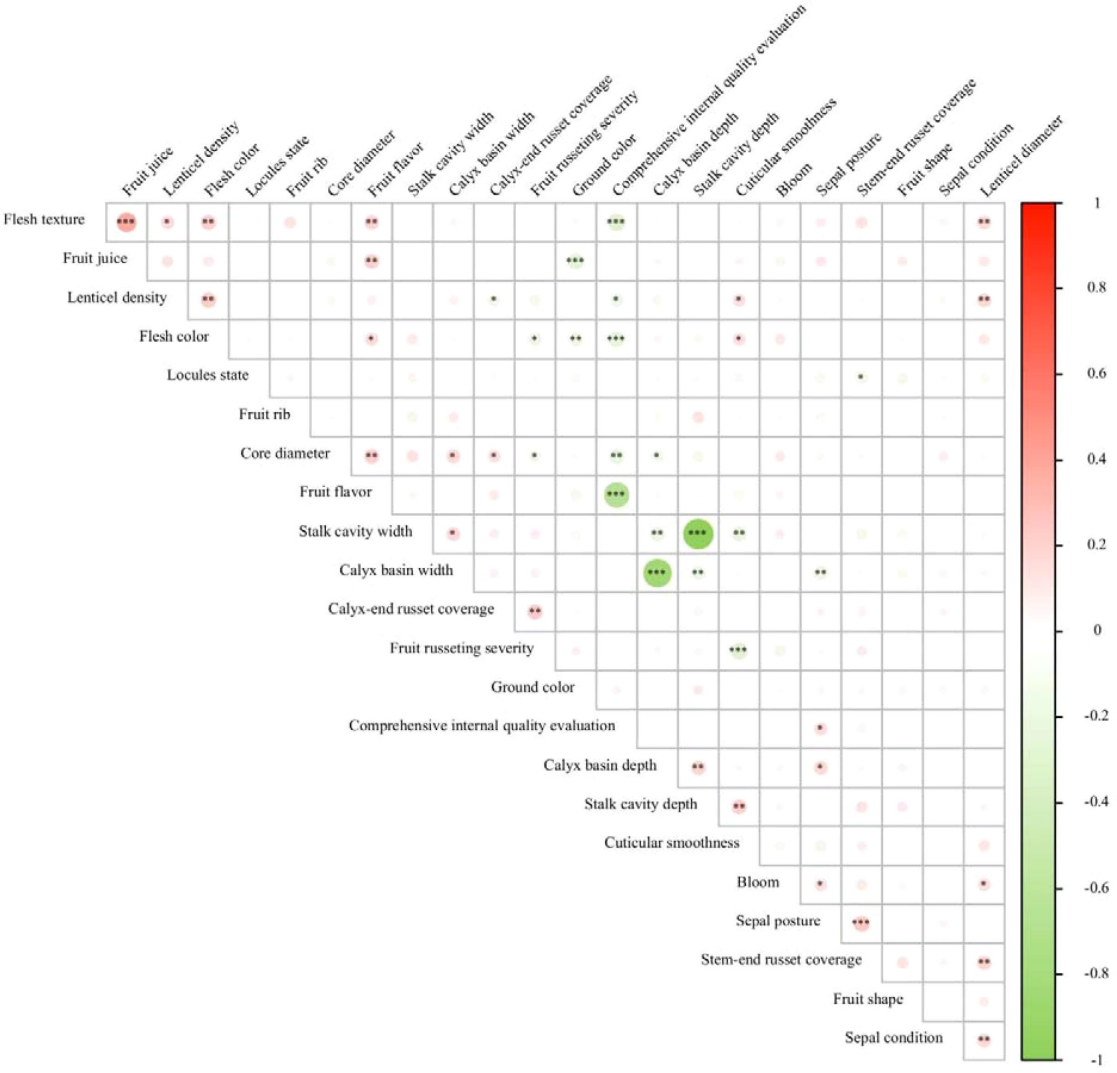
Correlation heatmap of fruit descriptive traits in *M. sieversii* germplasm resources. *:There are significant differences at the 0.05 level; **:There are significant differences at the 0.01 level; ****:There are significant differences at the 0.0001 level.

The correlation heatmap for the descriptive traits of *M. sieversii* germplasm resources is shown in **Figure 8**. Stalk cavity depth exhibited a highly significant positive correlation with calyx basin depth, and highly significant negative correlations with both stalk cavity width and calyx basin width. Stalk cavity width showed a highly significant negative correlation with calyx basin depth and a significant positive correlation with calyx basin width. Sepal posture was significantly positively correlated with calyx basin depth and highly significantly negatively correlated with calyx basin width. Calyx basin depth was highly significantly negatively correlated with calyx basin width and significantly negatively correlated with core diameter. Ground color showed highly significant negative correlations with both flesh color and fruit juice content. Calyx-end russet coverage demonstrated a highly significant positive correlation with fruit russeting severity. Fruit russeting severity showed a highly significant negative correlation with cuticular smoothness. Flesh texture was highly significantly positively correlated with both fruit juice and fruit flavor, while being highly significantly negatively correlated with the comprehensive internal quality evaluation. Fruit juice was highly significantly positively correlated with fruit flavor. Fruit flavor showed a highly significant negative correlation with the comprehensive internal quality evaluation.

### 3.4 PCA of fruit phenotypic traits

The PCA of the fruit phenotypic traits of *M. sieversii* germplasm resources is presented in **Table 4**. The twelve principal components extracted from the 31 phenotypic traits collectively accounted for 68.43% of the total variance, capturing the majority of the information from the original variables. The first principal component (PC1), with a contribution rate of 12.88%, was primarily associated with fruit size, as indicated by high loading values for fruit weight, fruit longitudinal and transverse diameters. The second principal component (PC2), accounting for 8% of the variance, was linked to fruit flavor quality, with major contributions from flesh texture, comprehensive internal quality evaluation, and fruit flavor. The third principal component (PC3), contributing 7.12% of the variance, was characterized as a stalk cavity-related factor, driven by stalk cavity depth and width. The fourth principal component (PC4) explained 6.87% of the variance and was identified as a pedicel-related factor, heavily influenced by pedicel length and diameter. The fifth principal component (PC5), with a contribution rate of 5.66%, represented a calyx basin-related factor, primarily associated with calyx basin depth and width. The contribution rates of the remaining principal components were all below 5%. In summary, traits such as fruit weight, fruit longitudinal and transverse diameters, flesh texture, comprehensive internal quality evaluation, and fruit flavor were the primary factors contributing to the phenotypic variation observed in the fruit of *M. sieversii* germplasm resources.

**Table 4:** Principal component analysis of fruit phenotypic traits in *M. sieversii* germplasm resources.

Traits	Principal content											
	PC1	PC2	PC3	PC4	PC5	PC6	PC7	PC8	PC9	PC10	PC11	PC12
Pedicel length	0.012	0.373	-0.078	-0.486	0.17	0.002	0.219	-0.298	0.239	0.022	-0.044	0.168
Pedicel diameter	0.407	-0.061	0.127	0.513	-0.026	0.002	-0.268	0.264	-0.26	-0.152	0.071	-0.008
Fruit weight	0.88	0.101	0.294	-0.017	0.046	-0.015	-0.119	-0.097	0.046	-0.086	-0.03	-0.037
Fruit longitudinal diameters	0.864	0.133	0.309	-0.145	0.094	-0.031	-0.213	-0.089	0.036	-0.014	0.058	0.018
Fruit transverse diameter	0.871	0.014	0.226	0.028	-0.096	-0.073	-0.105	-0.070	0.090	-0.128	-0.147	-0.097
Fruit index	0.180	0.252	0.207	-0.359	0.364	0.056	-0.254	-0.053	-0.081	0.194	0.382	0.205
Flesh firmness	-0.320	0.323	-0.016	0.265	0.043	0.128	0.439	-0.099	-0.018	-0.021	0.271	0.129
SSC	-0.234	-0.108	-0.057	-0.155	0.262	0.191	-0.413	0.342	0.117	0.105	0.181	0.048
Stalk cavity depth	0.502	0.005	-0.633	-0.174	-0.385	0.237	0.077	-0.041	-0.028	-0.050	0.138	0.013
Stalk cavity width	-0.499	-0.009	0.634	0.173	0.391	-0.240	-0.090	0.042	0.032	0.042	-0.132	-0.024
Sepal condition	0.085	0.119	0.135	0.036	-0.077	0.261	0.251	-0.180	-0.194	0.290	-0.267	0.166
Sepal posture	0.236	-0.035	0.045	0.360	0.179	0.161	0.435	0.171	0.282	-0.041	0.250	-0.048
Calyx basin depth	0.329	-0.164	-0.518	0.405	0.464	-0.003	-0.077	-0.201	0.079	0.165	0.015	0.067
Calyx basin width	-0.279	0.173	0.554	-0.433	-0.457	0.029	0.118	0.104	-0.095	-0.192	-0.019	-0.101
Locules state	-0.132	-0.041	-0.026	-0.050	0.046	-0.321	-0.184	-0.580	0.171	-0.177	0.065	0.198
Fruit shape	0.185	0.023	-0.073	0.146	-0.070	0.038	0.040	0.285	-0.450	0.230	-0.130	0.401
Ground color	-0.010	-0.362	-0.106	-0.226	-0.144	0.106	-0.060	0.333	0.463	-0.037	-0.008	0.097
Calyx-end russet coverage	-0.065	-0.031	0.268	0.154	-0.081	0.331	-0.082	-0.134	0.083	0.495	0.304	-0.412
Fruit russeting severity	0.053	-0.206	0.347	0.267	-0.372	-0.020	0.048	-0.067	0.392	0.276	0.000	0.174
Stem-end russet coverage	0.388	0.270	0.154	0.077	-0.018	0.408	0.109	0.096	0.215	-0.030	-0.131	-0.072
Bloom	-0.036	0.045	0.096	0.146	0.327	0.395	0.123	0.002	-0.029	-0.627	0.024	-0.067
Cuticular smoothness	0.130	0.219	-0.359	-0.442	0.111	-0.002	-0.016	0.052	-0.070	0.201	-0.253	-0.468
Fruit rib	0.006	0.054	-0.105	-0.118	-0.355	-0.121	-0.099	-0.041	-0.192	-0.193	0.527	0.003
Lenticel diameter	0.266	0.429	0.151	-0.178	0.187	0.167	0.189	0.098	-0.068	0.062	-0.032	0.284
Lenticel density	-0.042	0.438	-0.110	-0.219	0.140	-0.223	-0.057	0.468	0.211	-0.013	0.013	0.142
Core diameter	-0.310	0.187	0.127	-0.096	0.018	0.524	-0.274	-0.193	-0.232	0.019	0.074	-0.001
Flesh color	-0.171	0.496	-0.144	0.127	0.299	-0.087	0.035	0.028	0.001	-0.068	-0.022	-0.301
Flesh texture	0.079	0.593	-0.005	0.133	-0.170	-0.244	0.074	0.119	0.194	0.120	0.203	-0.031
Fruit juice	0.179	0.428	-0.005	0.280	-0.110	-0.475	0.111	0.039	-0.199	0.093	0.070	-0.149
Fruit flavor	-0.271	0.503	-0.084	0.405	-0.284	0.138	-0.303	-0.122	0.138	-0.091	-0.161	0.039
Comprehensive internal quality evaluation	0.263	-0.556	0.214	-0.265	0.229	-0.174	0.426	0.009	-0.186	0.033	0.167	-0.123
Eigenvalue	3.992	2.479	2.207	2.13	1.753	1.528	1.395	1.289	1.22	1.17	1.036	1.015
Contribution (%)	12.88	8.00	7.12	6.87	5.66	4.93	4.50	4.16	3.94	3.78	3.34	3.27
Cumulative contribution (%)	12.88	20.87	27.99	34.86	40.52	45.45	49.95	54.11	58.04	61.82	65.16	68.43

### 3.5 Cluster analysis of fruit phenotypic traits

**Figure 9:**
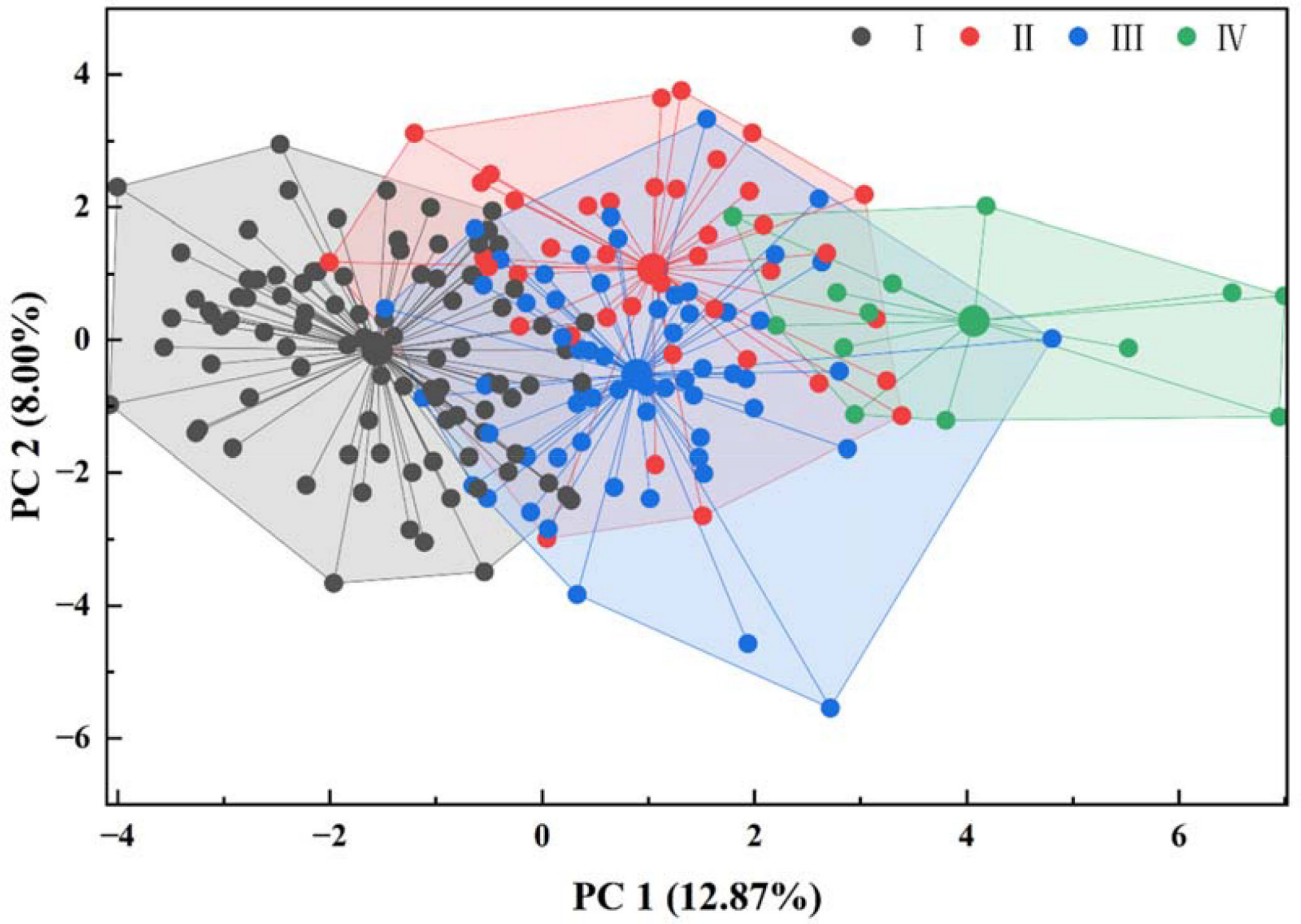
Clustering analysis of fruit phenotypic traits in *M. sieversii* germplasm resources.

The cluster analysis of fruit phenotypic traits for the *M. sieversii* germplasm resources is shown in **Figure 9**. Based on K-means cluster analysis of the 31 phenotypic traits, the 210 accessions were grouped into four distinct clusters. Cluster I comprised 96 accessions, including GB7, GD9, GD11, GD12, GD13, GJS3, ML24, XY6, XY10, and XY92, accounting for 45.71% of the total population. This cluster exhibited the highest mean values for SSC and fruit flavor (acidity) but the lowest mean value for comprehensive internal quality evaluation, indicating a notably “sour taste”. Furthermore, it had the lowest mean values for fruit weight, fruit longitudinal and transverse diameters, signifying small fruit size. Therefore, this cluster was characterized as small-fruited, high-acidity resources. Cluster II included 40 accessions, such as CK10, CK18, EM52, HDM17, HDM21, HDM22, HDM35, YA4, YA32, and YA36. It displayed the highest mean value for pedicel length and the lowest mean SSC. While having a medium fruit size, it showed the highest mean fruit index, indicating a “subglobose” fruit shape. This cluster was defined as resources with the specific trait of long pedicels. Cluster III consisted of 61 accessions, including EM4, EM21, GALS3, GJS24, HDM47, LF6, LF10, XY82, YA9, and YA19. Its fruit size was highly similar to that of Cluster II. However, it had the lowest mean values for pedicel length and fruit index, the shortest intra-cluster distances, and the highest inter-accession similarity. This cluster represents oblate-fruited, short-pedicel resources. Cluster IV contained only 13 accessions (CK3, EM3, GB18, GJS2, GK3, HDM30, HDM34, HDM37, HONG3, XY62, YG1, YG4, YJ22), representing 6.2% of the total population. Phenotypically, this cluster was distant from the other three and exhibited the greatest intra-cluster dispersion, indicating its distinctiveness. It had the highest mean values for fruit weight, fruit longitudinal and transverse diameters, indicating large fruit size. It also showed the highest mean values for calyx-end russet coverage, stem-end russet coverage, and fruit russeting severity, indicating “extensive” fruit surface russeting. Furthermore, it displayed the highest mean values for flesh texture, fruit juice, and comprehensive internal quality evaluation, coupled with the lowest mean value for fruit flavor (acidity), suggesting “crisp and tender” flesh, “high” juiciness, a “balanced sweet-sour” flavor, and excellent eating quality. Consequently, this cluster represents large-fruited resources with superior flavor quality. It serves as valuable and unique research material specifically for studying extensive russeting and constitutes a relatively rare and precious germplasm resource.

## 4 Discussion

This study provides a systematic evaluation of 31 fruit traits across 210 *M. sieversii* accessions from seven natural populations, revealing substantial phenotypic diversity. The results indicate extensive variation in key traits such as fruit weight, pedicel length, and flavor quality. These findings not only confirm the potential of *M. sieversii* as a valuable gene pool (Cornille et al., 2013; Duan et al., 2017; Gao et al., 2020) but also offer a direct basis for its efficient utilization. Frequency distribution and statistical analysis revealed that the fruit weight of the 210 accessions ranged from 6.88 to 44.33 g, exhibiting a broad distribution and a high coefficient of variation (38.24%). The continuous spectrum of fruit weight, from very small to large, provides a basis for breeding apples with diverse fruit types (Xu et al., 2025). Pedicel length varied between 6.50 and 36.29 mm (CV: 31.03%), and pedicel diameter ranged from 0.8 to 2.68 mm (CV: 20.01%). Combined with correlation and cluster analyses, pedicel length and diameter showed a highly significant negative correlation and substantial variation among the *M. sieversii* accessions, identifying them as one of the core traits driving population differentiation. The high CV observed for these three quantitative fruit traits reflect their considerable dispersion. Generally, a higher CV indicates greater disparity in a trait among different resources (Tian et al., 2024) and richer genetic diversity. The external quality of fruit is primarily determined by fruit size, color, and shape (Wu et al., 2024; Gao, 2025). Superior external quality not only serves as a key factor stimulating consumption but also critically determines the commercial value of the fruit (Zhao et al., 2022). The ground color of the fruit among the 210 accessions encompassed seven types: “pale green”, “yellow-green”, “green”, “green-yellow”, “pale yellow”, “yellow”, and “yellowish white”, predominantly featuring shades of “green” and “yellow”, with a minimal presence of “yellowish white”. Similarly, the flesh color included seven categories: “white”, “yellowish white”, “greenish white”, “pale yellow”, “yellow”, “yellow-green”, and “light red”. The majority exhibited whitish hues such as “greenish white” and “yellowish white”, while “pale yellow”, “yellow-green”, and “light red” were rare. *M. sieversii* displays remarkable diversity in both fruit size and ground color, making it a valuable resource for improving external fruit quality in modern breeding programs. The internal quality of fruit encompasses attributes such as firmness, juice content, SSC, and flavor (Zhen, 2024; Chen et al., 2024; Wang et al., 2025). Flesh firmness among the 210 accessions ranged from 2.89 to 13.3 kg/cm², with a CV of 22.49%. The texture spanned a wide spectrum, from “soft and spongy”, “mealy”, “crisp and tender”, to “firm and crisp”, and “hard”, demonstrating substantial and diverse variation. Accessions with high firmness can serve as parental materials for breeding varieties with enhanced storage tolerance (Zhang et al., 2020; Lv et al., 2021). SSC ranged from 9.06% to 20.77%, showing a relatively narrow variation. Fruit flavors included “mildly sweet”, “sweet-sour”, “balanced sweet-sour”, “sour-sweet”, “sour”, and “very sour”. “Sour” was the most prevalent flavor (48.57%), followed by “very sour” (22.86%). Sweet-leaning flavors such as “mildly sweet” and “sweet-sour” were less common, indicating a predominance of sourness. Notably, resources with “very sour” flavors can be cultivated as functional materials, which aligns with findings from previous studies (Ran et al., 2023). Therefore, the *M. sieversii* germplasm resources exhibit rich genetic diversity in both external and internal fruit quality traits, establishing them as a precious gene pool for modern apple breeding.

The results of this study indicate significant correlations among multiple phenotypic traits in *M. sieversii* fruit. Among the quantitative traits, fruit weight showed highly significant positive correlations with both fruit longitudinal and transverse diameters, which aligns with the expected growth patterns of fruit and is consistent with the findings of Tian et al. (2025). Concurrently, fruit weight was also highly significantly positively correlated with the fruit index, implying that larger fruits tend to have a relatively greater longitudinal diameter and a more elongated shape. Conversely, this finding corroborates the results from the frequency distribution analysis, where fruits weighing 10–20 g (small-sized) were most prevalent, and the predominant fruit shape was “oblate”. This phenomenon may arise from an unequal spatial allocation between cell division and expansion during fruit development (Ding et al., 2018). A highly significant negative correlation was observed between fruit weight and flesh firmness, indicating that smaller fruits tended to exhibit greater firmness. This phenomenon may be attributed to the hardness tester probe more readily contacting the core when measuring small fruits, potentially leading to an overestimation of firmness. This observation requires further verification. Among the qualitative traits, stalk cavity depth exhibited a highly significant negative correlation with stalk cavity width, indicating that deeper stalk cavities were associated with narrower widths. Similarly, calyx basin depth showed significant negative correlations with both calyx basin width and core diameter, suggesting that deeper calyx basins were linked to narrower widths and smaller cores. Based on the correlation analysis among flesh texture, juice, flavor, and comprehensive internal quality evaluation, it was observed that “firmer and crisper” or “hard” flesh textures were associated with higher juice content and a more acidic flavor, consequently resulting in “fair” or “poor” comprehensive internal quality scores. Ground color demonstrated highly significant negative correlations with both flesh color and fruit juice. This implies that fruits with ground colors closer to “pale yellow” or “yellow” tended to have flesh colors approaching “white” or “yellowish white”, along with lower juice. Conversely, ground colors closer to “yellow-green” or “green” tended to exhibit flesh colors approaching “yellow-green” or “greenish white”, along with higher juice. These findings suggest a significant coordinated pattern between the transition of the external ground color from “green” to “yellow” and the internal flesh color transition from “greenish white” to “white” or “yellowish white”. This may reveal an underlying biological logic of spatially coordinated pigment metabolism regulation during fruit ripening, warranting further in-depth investigation.

Currently, PCA and cluster analysis are widely employed methods for evaluating fruit quality (Li et al., 2021; An et al., 2025). PCA reduces the dimensionality of multiple correlated variables in the original dataset into a smaller set of variables through linear transformation, while maximally retaining the principal information contained within the original data (Šamec et al., 2016). This study found that the eigenvalues of the first 12 principal components for the fruit phenotypic traits were greater than 1, cumulatively explaining 68.43% of the total variation. Among these, the first four principal components accounted for 34.86% of the cumulative contribution, representing the primary dimensions underlying the phenotypic diversity of *M. sieversii* fruit (Zhao et al., 2020). The complex set of 31 traits can thus be simplified into a few comprehensive factors with biological significance. Specifically, the observed variation primarily revolves around fruit size (Zhang et al., 2021), flavor quality, cavity morphology, and pedicel characteristics. This simplification provides efficient evaluation dimensions for the subsequent rapid screening of germplasm resources and the construction of a core collection. Cluster analysis groups resources with similar characteristics, thereby enhancing the understanding of similarities and differences among accessions. In this study, K-means cluster analysis partitioned the 210 *M. sieversii* accessions into four phenotypically distinct clusters. Cluster I was characterized as small-fruited, high-acidity resources; Cluster II as long-pedicel resources; Cluster III as oblate-fruited, short-pedicel resources; and Cluster IV as a unique group of large-fruited resources with extensive russeting and superior fruit flavor quality. This classification objectively reveals the discrete structure of fruit traits, providing a framework for the evaluation, conservation, and utilization of these resources. Combining principal component and cluster analyses revealed that PC1 (fruit size) is the primary driver of phenotypic differentiation in *M. sieversii*. It effectively separated Cluster IV, which consists of uniquely large-fruited accessions. This finding corroborates that fruit size is often under strong selection in cultivated apples and is a trait highly prone to discrete variation. Dimensions such as PC2 (flavor quality) and PC4 (pedicel characteristics) collectively drive the further differentiation within the small-fruited (Cluster I) and medium-fruited (Clusters II and III) groups. The partial differentiation of Cluster I (small-fruited, high-acidity resources) and Cluster III (medium-fruited, short-pedicel resources) is driven by PC2. Cluster I exhibits positive loadings on PC2, characterized by sour flavor and lower comprehensive internal quality evaluation, representing a typical phenotype of *M. sieversii* germplasm. In contrast, Cluster III shows negative loadings on PC2, associated with a sweet-sour flavor and higher comprehensive internal quality evaluation. Clusters II and III, while highly similar on PC1, are primarily differentiated by PC4. Cluster II displays negative loadings on PC4, featuring thin and long pedicels that may facilitate fruit pendency. Conversely, Cluster III shows positive loadings on PC4, characterized by thick and short pedicels that likely provide stronger mechanical support for the fruit. Therefore, when constructing a core collection of *M. sieversii* germplasm, it is essential to ensure adequate representation of all four clusters within the phenotypic space defined by PC1 through PC4.

## 5 Conclusion

Based on the comprehensive results, the majority of the 210 *M. sieversii* accessions are characterized as small, oblate fruits with green-yellow skin, smooth and ribbed surfaces, and a predominantly sour flavor. Their diversity is structured through the combination and variation of several core and relatively independent dimensions: fruit size, flavor quality, cavity morphology, and pedicel characteristics. Ultimately, these accessions can be categorized into four distinct groups: small-fruited, high-acidity germplasm; germplasm with long-pedicel traits; germplasm with short-pedicel traits; and a unique large-fruited germplasm with superior flavor quality.

## Data Availability

The original contributions presented in the study are included in the article/supplementary material. Further inquiries can be directed to the corresponding author.
