# Correction: Evaluation of fruit phenotypic diversity in 210 *Malus sieversii* germplasm resources

**DOI:** 10.3389/fpls.2026.1887348

**Published:** 2026-05-29

**Authors:** Bian Ran, Ling Zhu, Shengjun Zhang, Junnan Jian, Fang Tian, Shilei Dong, Shimin Tang, Xuechao Zhang

**Affiliations:** Institute of Agricultural Sciences of Ili Kazakh Autonomous Prefecture, Key Laboratory of Crop Breeding and Quality Testing, Yili Prefecture Agriculture and Rural Affairs Bureau, Yili, Xinjiang, China

**Keywords:** diversity, Fruit, Germplasm resources, Malus sieversii, phenotype

The funder “Modern Agriculture and Animal Husbandry Industry Technology Enhancement Project of Ili Prefecture (Jiangsu Academy of Agricultural Sciences Yili Branch)” was erroneously omitted.

The original version of this article has been updated.

